# The Induction of Vascular Endothelial Growth Factor by Ultrafine Carbon Black Contributes to the Increase of Alveolar-Capillary Permeability

**DOI:** 10.1289/ehp.7457

**Published:** 2005-01-04

**Authors:** Chih-Ching Chang, Hui-Fen Chiu, Yih-Shyuan Wu, Yi-Chih Li, Mei-Ling Tsai, Chen-Kuo Shen, Chun-Yuh Yang

**Affiliations:** ^1^Graduate Institute of Public Health,; ^2^Department of Pharmacology, and; ^3^Graduate Institute of Medicine, Kaohsiung Medical University, Kaohsiung, Taiwan; ^4^Department of Physiology, College of Medicine, National Cheng Kung University, Tainan, Taiwan

**Keywords:** intratracheal instillation, permeability, reactive oxygen species, ultrafine carbon black, vascular endothelial growth factor, VEGF

## Abstract

Ultrafine carbon black (ufCB) can cause proinflammatory response and increase alveolar-capillary permeability. However, the mechanism underlying the increased permeability is not well characterized. Vascular endothelial growth factor (VEGF) is originally recognized as a vascular permeability factor. Oxidative stress generated by hydrogen peroxide (H_2_O_2_) stimulates VEGF gene expression. The purpose of this study was to explore the role of VEGF in ufCB-induced alveolar-capillary permeability. Intratracheal instillation of 200 μg ufCB in mice caused a significant and sustained increase of total proteins in bronchoalveolar lavage (BAL) fluid, with the maximal increase at 21 hr postinstillation. The influx of neutrophils did not significantly increase until 16 hr. It reached the highest level at 21 hr and returned to the basal level by 42 hr. Tumor necrosis factor-α was significantly elevated only at 4 hr. ufCB induced significant increases of VEGF in BAL fluid throughout the study period, with the peak increase at 16 hr. The nonsecreted isoform VEGF188 was not altered after 16 hr of exposure to ufCB. Moreover, there was a strong correlation between VEGF and total proteins in BAL fluid (*R*^2^ = 0.7352, *p* < 0.01). *In vivo* study supported the role of reactive oxygen species (ROSs) in ufCB-induced VEGF release and protein leakage. The involvement of ROSs was strengthened by the fact that interventions with *N*-acetyl-cysteine prevented ufCB-induced generation of ROSs and VEGF *in vitro*. Our study for the first time demonstrates that ufCB induces the production of VEGF, which is associated with the increase of alveolar-capillary permeability. The induction of VEGF by ufCB acts through an ROS-dependent pathway.

Epidemiologic studies have shown that exposure to PM_10_ (particulate matter ≤ 10 μm in diameter) is associated with decreased lung function and increased emergency hospital admissions for respiratory diseases (Atkinson et al. 1999; Dusseldorp et al. 1995). Exposure to PM_2.5_ (PM ≤2.5 μm in diameter) is related to increases in all-cause, cardiopulmonary, and lung cancer mortality (Laden et al. 2000; Pope et al. 2002). In ambient air, carbon-centered ultrafine particles (PM ≤0.1 μm in diameter) are often generated during internal combustion processes and constitute most fine or coarse particulate matter (Oberdorster and Utell 2002). In adults with a history of asthma, ultra-fine particles play a more significant role in producing detrimental respiratory effects than does PM_10_ (Peters et al. 1997). Use of asthma medication is associated with an increase in the number concentration of ultrafine particles (Von Klot et al. 2002). These findings indicate that exposure to ultrafine particles can increase the susceptibility of individuals with preexisting diseases to particle health effects.

Our knowledge of ultrafine-particle–induced lung injury has drawn on *in vivo* studies of ultrafine materials, such as ultrafine titanium dioxide (ufTiO_2_; 20 nm in diameter) and ultrafine carbon black (ufCB; 14 nm in diameter). ufTiO_2_ particles provoke influxes of polymorphonuclear cells, enhance protein exudation into alveolar space, prolong particle interstitial retention, and increase lung lymph node burden to a greater extent than do fine TiO_2_ particles (~ 250 nm in diameter) in rats (Ferin et al. 1992; Oberdorster et al. 1992). Significantly greater increases in neutrophil influx and total protein in bronchoalveolar lavage (BAL) fluid are observed after intratracheal instillation of ufCB, compared with carbon black (CB; 250 nm in diameter) (Li et al. 1996). This may be due to the relatively large particle number and surface area per unit of deposited ufCB mass (Donaldson et al. 1998; Oberdorster 1996; Stone et al. 1998). A recent study has found that the time response of the increase in total proteins after ufCB instillation is very similar to that of neutrophil influx and that significant increases in tumor necrosis factor-α (TNF-α) production and total proteins are present 6 hr after instillation of ufCB (Li et al. 1999). However, another study has found that ufCB induces a significant influx of neutrophils but only a moderate and non-significant increase in total proteins (Dick et al. 2003). These studies do not elaborate on the attributes that may contribute to the increase of alveolar-capillary permeability. As a result, the mechanism of ufCB-induced alveolar-capillary permeability has yet to be elucidated.

Vascular endothelial growth factor (VEGF) is an endothelial-cell–specific mitogen and the most potent vascular growth factor found to date. It induces vasculogenesis from pre-endothelial cells and angiogenesis from pre-existing capillaries (Carmeliet 2000; Ferrara 1999). In addition, VEGF had been identified as a vascular permeability factor before its discovery as an endothelial cell mitogen (Senger et al. 1983, 1993). By delivering E1^−^ adeno-virus vector (AdVEGF165) to the mouse respiratory epithelium, the overexpression of VEGF165 isoform significantly increased pulmonary vascular permeability 24 hr after administration (Kaner et al. 2000). In mice, time-dependent increases of total lung VEGF mRNA are associated with neutrophil influx and increases in total proteins in BAL fluid after exposure to lipopolysaccharide (LPS) (Karmpaliotis et al. 2002).

Reactive oxygen species (ROSs) have been shown to stimulate VEGF production (Chua et al. 1998; Kuroki et al. 1996). ufCB is capable of depleting supercoiled plasmid DNA and increasing 2′,7′-dichlorofluorescein (DCF) fluorescence in a cell-free system, indicating the existence of surface free radical activity (Dick et al. 2003; Stone et al. 1998; Wilson et al. 2002). After incubation with human monocytes, ufCB can further stimulate the production of ROS and increase oxidative stress (Wilson et al. 2002). As a result, it is reasonable to hypothesize that ufCB can cause oxidative stress and increase the production of VEGF in lung tissue.

The purpose of this study is to investigate the role of VEGF in ufCB-induced lung injury. Accordingly, the study explores the kinetic of VEGF production and its influence on alveolar-capillary permeability after exposure to ufCB. In addition, the involvement of ROSs in ufCB-induced production of VEGF is investigated.

## Materials and Methods

### Animals.

Male ICR mice were purchased from the animal center at National Cheng Kung University (Tainan, Taiwan). All animal experiments were conducted according to the National Institutes of Health (NIH) guidelines (NIH 1996). The procedures were approved by the Animal Care and Use Committee of Kaohsiung Medical University.

### Particle preparation.

ufCB (Printex 90; diameter, 14 nm; surface area, 253.9 m^2^/g; Degussa, Frankfurt, Germany) was weighed, ground, and suspended in phosphate-buffered saline (PBS; pH 7.4). The suspension was then sonicated (model W-250; Branson, Danbury, CT, USA) for 20 min (20 W) before adjusting it to the final concentration of 2μg/μL.

### Intratracheal instillation and broncho-alveolar lavage.

Male ICR mice were housed two per cage in the Kaohsiung Medical University animal center with a schedule of 14 hr of light (0500–1900 hr) and with room temperature control (24 ± 1°C). At the age of 5 weeks and weight 25–30 g, the mice were anesthetized by intraperitoneal injection with 1.25% citosol (Kyorin Pharmaceuticals, Tokyo, Japan) and instilled with an intratracheal application with 100 μL ufCB (200 μg/100 μL/mouse) or LPS (1 μg/μL, 100 μg/100 μL/mouse; Sigma, St. Louis, MO, USA). Sterile PBS (100 μL) was used as our vehicle control. At 4, 16, 21, and 42 hr after intratracheal instillation, the mice were euthanized by intraperitoneal injection with 2.5% citosol. The lung was exposed and the trachea was cannulated for lavaging the broncho-alveolar space two times with 1 mL PBS. The BAL samples were centrifuged at 300 × *g* for 10 min. The cell pellets were resuspended in 1 mL RPMI 1640 medium. The primary lavage recovered was separated from subsequent lavage and used for the measurement of total proteins, TNF-α, and VEGF in BAL fluid. For Western blotting analysis, the unlavaged lungs were removed, minced into small pieces, snap-frozen in liquid nitrogen, and stored at –80°C until analyzed for VEGF.

Initially, there were eight mice for each time point (except *n* = 4; *n* = 3 for 42-hr exposure group). To conclude that the peak increase of VEGF appeared before that of total proteins, a second study was conducted with a total of eight mice for the 16-hr exposure group (*n* = 4) and 10 mice for the 21-hr exposure group (*n* = 5). In total, for the 4-hr group, *n* = 4; 16-hr group, *n* = 8; 21-hr group, *n* = 9; 42-hr group, *n* = 3.

To further study the effects of *N*-acetyl-cysteine (NAC; a glutathione precursor and antioxidant) on ufCB-induced productions of VEGF and total proteins, mice were injected with NAC (320 mg/kg) intraperitoneally 2 hr before ufCB instillation. Mice were sacrificed after 16 hr of exposure to ufCB.

### Analysis of BAL leukocytes.

An aliquot of the cell suspension was examined for total number of white blood cells using a hemo-cytometer (Paul Marienfeld GmbH, Lauda-Koenigshofen, Germany). In addition, cytospin slides were prepared using 3 ×10^4^ cells centrifuged at 180 ×*g* for 5 min onto glass slides. After Liu stain, differential cell counts of neutrophils, macrophages, and lymphocytes were determined by counting 400 cells under a microscope (magnification, 400×; Olympus, Tokyo, Japan).

### Quantitation of total proteins, VEGF, and TNF-αin BAL fluid.

Total proteins in BAL fluid were measured using a Bio-Rad protein assay kit, according to the manufacturer’s manual (Bio-Rad, Hercules, CA, USA). Enzyme-linked immunosorbent assays (ELISAs) were adopted to measure mouse VEGF and TNF-α and performed according to manufacturer’s protocols (R&D systems, Chantilly, VA, USA). VEGF ELISA detects mature VEGF containing 120 and 164 amino acid residues (secreted VEGF). The optical density of each sample was determined at a wavelength of 450 nm and a reference wavelength of 540 nm by a microplate reader (OpsysMR, DYNEX, Minneapolis, MN, USA).

### Western blot analysis.

Lung tissues were grounded in lysis buffer containing 20 mM Tris, 150 mM NaCl, 1 mM EDTA, 1% Nonidet P-40, 1 mM Na_3_VO_4_, and 100 μg/mL phenylmethylsulfonyl fluoride. After being centrifuged (Microfuge R, Beckman, CA, USA) at 7,500 × *g*, 4°C for 20 min, the supernatant was collected for measuring protein contents using a Bio-Rad protein assay kit. Bovine serum albumin (BSA) was used as the standard. Equal amounts of tissue homogenate (100 μg) were loaded to each lane on a 12.5% sodium dodecyl sulfate–polyacrylamide gel. Molecular weight markers of proteins were loaded simultaneously. After electrophoresis at 4°C for 6 hr, separated proteins in the gel were transferred onto a polyvinyldifluoride membrane. The membrane containing transferred proteins was treated with 5% nonfat milk in PBS Tween to block nonspecific binding. After incubations with first and secondary antibodies, the membrane was treated with electrogenerated chemiluminescence reagents to enhance signals. Finally, exposure of the membrane to the light-imaging film (Kodak, Boston, MA, USA) visualized the bands. The protein abundance of β-actin was detected as an internal control. Densitometry with a computerized image analyzer was used to quantify the results (GelDoc-IT System; UVP, Upland, CA, USA).

Two primary antibodies were used in this study: VEGF (1:1,500; Santa Cruz Biotechnology, Santa Cruz, CA, USA) and β-actin (1:10,000; Chemicon, Temecula, CA, USA). VEGF antibody can react with all three VEGF isoforms in mice.

### Histochemistry.

Shortly after the mice were sacrificed, the lungs were fixed by instillation of 10% formaldehyde at a pressure of 25 cm H_2_O for 5 min. The specimens were then merged in 4% paraformaldehyde at 4°C for 48 hr. After fixation and washing, the specimens were embedded in paraffin wax. Sections 5 μm thick from each paraffin block were expanded on slides and stained with hematoxylin and eosin (H&E; Vector Labs, Burlingame, CA, USA). The histopathologic analysis was performed using a microscope with cooled charged couple device (magnification, 1,000×; Olympus).

### Measurement of ufCB-induced ROS production in THP-1 and A549 cells by DCFH-DA.

ROS production was determined using 2′,7′-dichlorofluorescein diacetate (DCFH-DA), which was an oxidant-sensitive fluores-cent probe. Inside the cell, the probe was deacetylated by esterases and formed H_2_DCF, which was oxidized by ROS to DCF, a highly fluorescent compound (LeBel et al. 1992).

THP-1, a human monocyte cell line, was obtained from American Type Culture Collection (Manassas, VA, USA) and cultured in RPMI 1640 medium supplemented with 10% fetal bovine serum (FBS), 1% penicillin/streptomycin, and 1% L-glutamine in a 37°C humidified incubator supplied with 5% CO_2_. Before treatment, cells (7 × 10^5^) were loaded with 20 μM (final concentration) DCFH-DA for 1 hr. The cells were then treated for 4 hr with 100 μg/mL ufCB or culture medium, with or without 20 mM NAC pretreatment for 1 hr. Cell viability was checked using trypan blue exclusion test and was > 95% in all groups. The DCF fluorescence intensity was determined by a FLUOStar OPTIMA (BMG LabTech, Offenburg, Germany). The arbitrary value of emitted DCF fluorescence intensity would reflect the extent of ROS production in THP-1 cells.

A549, a human lung adenocarcinoma cell line/type II alveolar epithelial cell, was obtained from American Type Culture Collection and cultured in Dulbecco modified Eagle medium supplemented with 10% FBS, 1% penicillin/streptomycin, and 1% L-glutamine in a 37°C humidified incubator supplied with 5% CO_2_. Cells (7 × 10^5^) adhering to the culture dish were treated for 4 hr with 100 μg/mL ufCB or culture medium, with or without 20 mM NAC pretreatment for 1 hr. At the end of incubation periods, cells were washed with PBS and immediately detached with trypsin/EDTA, and then incubated for 30 min with 20 μM (final concentration) DCFH-DA at 37°C. The cells were washed twice with PBS, followed by analysis on a FACSCalibur flow cytometer (Becton Dickinson Biosciences, San Jose, CA, USA). The fluorescence was plotted against the number of cells. The extent of ROS production in A549 cells was indicated as the ratio of mean fluorescence intensity of treated cells to PBS control.

### Quantitation of ufCB-induced VEGF production in THP-1 and A549 cells.

THP-1 and A549 cells (7 × 10^5^) were treated for 4 hr with 100 μg/mL ufCB in serum-free culture media, with or without 20 mM NAC pre-treatment for 1 hr. To determine cellular VEGF production, cultured cells were treated with lysis buffer containing 1% Nonidet P-40, 0.5% sodium deoxycholate, 10% sodium dodecyl sulfate, 1 mM Na_3_VO_4_, and 100 μg/mL phenylmethylsulfonyl fluoride. Cellular VEGF concentrations were assessed by human VEGF ELISA kit (R&D Systems, Minneapolis, MN, USA) and normalized to protein contents.

### Data analysis and statistical evaluation.

All data are expressed as means ± SE. Comparisons between groups were performed using one-way analysis of variance followed by a pairwise comparisons in the means model of SPSS (SPSS, Chicago, IL, USA). The relationship between cytokine production and total proteins was evaluated using the coefficient of determination (*R*^2^). In all cases, a *p*-value of <0.05 was considered statistically significant.

## Results

### Time-dependent effect of ufCB-induced acute lung injury.

**Figure 1:**
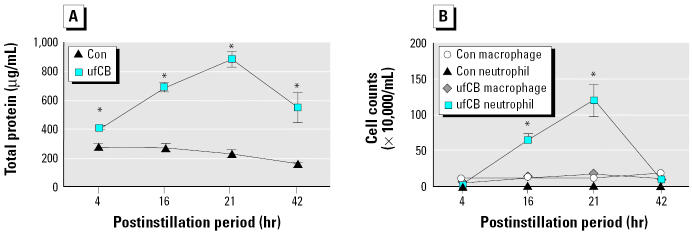
Time-dependent effect of ufCB (200 μg/mouse) on (*A*) total proteins and (*B*) cell counts. The value of each point is expressed as a mean ± SE of three to nine mice: 4 hr, *n* = 4; 16 hr, *n* = 8; 21 hr, *n* = 9; 42 hr, *n* = 3.
*Significantly different from the control (Con) of each time point (*p* < 0.01).

Total proteins in BAL fluid were measured as an indicator of alveolar-capillary permeability, and BAL leukocytes with differential counts were calculated as an indicator of inflammatory cell infiltrated in alveolar space. In conjunction, the amounts of TNF-α and VEGF in BAL fluid were measured. Our preliminary data had indicated that ufCB at 200 μg/mouse induced the greatest VEGF production and protein leakage. Accordingly, we used ufCB at 200 μg/mouse in this study. When compared with the controls, ufCB induced significant increases of total proteins in BAL fluid at 4, 16, 21, and 42 hr postinstillation (Figure 1A). Total proteins reached the maximal level at 21 hr and were still evident 42 hr later. However, ufCB induced a slower influx of BAL neutrophils, which became moderate but significant increase at 16 hr, maximal increase at 21 hr, and no measurable difference from the control group by 42 hr (Figure 1B). The data indicated that the increase of alveolar-capillary permeability started earlier than the increase in neutrophil influx. Moreover, the increased alveolar-capillary permeability sustained for a longer period than did neutrophil influx.

**Figure 2:**
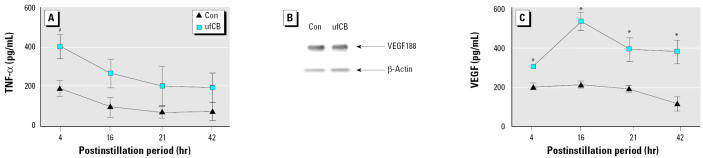
Time-dependent effect of ufCB (200 μg/mouse) on production of (*A*) TNF-α and (*C*) VEGF. The value of each point is expressed as a mean ± SE of three to nine mice. (*B*) Western blot analysis. The protein abundance of VEGF188 isoform in lung tissue is not altered after 16 hr of exposure to ufCB.
*p < 0.01 and ^#^*p* < 0.05 compared with the control at that time point.

Correspondingly, the time-dependent effects of ufCB on the production of TNF-α and VEGF in BAL fluid were determined. Four hours after instillation of ufCB, there was a significant increase of TNF-α in BAL fluid, compared with control group (*p* < 0.05). However, the marked increase of TNF-α at 4 hr decreased to a level with no difference from that of control group at 16 hr, and it further dropped at 21 hr and 42 hr (Figure 2A). As for VEGF, there were three VEGF isoforms (VEGF188, VEGF164, and VEGF120) with different molecular weights in mouse lung tissue (Shima et al. 1996). VEGF188 containing heparin-binding domain was considered a non-secreted form of VEGF. Western blotting was adopted to evaluate the changes of nonsecreted VEGF188. VEGF164 and −120, having no heparin-binding domain, were secreted forms and could be measured by ELISA. Western blot analysis showed that the expression of non-secreted VEGF188 was not significantly different from that of control (Figure 2B). The data from ELISA indicated that the secreted forms of VEGF in BAL fluid were significantly increased at 4, 16, 21, and 42 hr postinstillation. Most significantly, VEGF reached the highest level at 16 hr postinstillation (Figure 2C). These suggested that ufCB increased the production of TNF-α and secreted forms of VEGF in a time-dependent manner.

Taken together, there was a significant elevation of TNF-α , VEGF, and total proteins in BAL fluid at 4 hr postinstillation to ufCB. VEGF reached the maximal increase at 16 hr postinstillation (Figure 2C), which was earlier than that of total proteins at 21 hr (Figure 1A).

### BAL cytology and histopathology.

**Figure 3:**
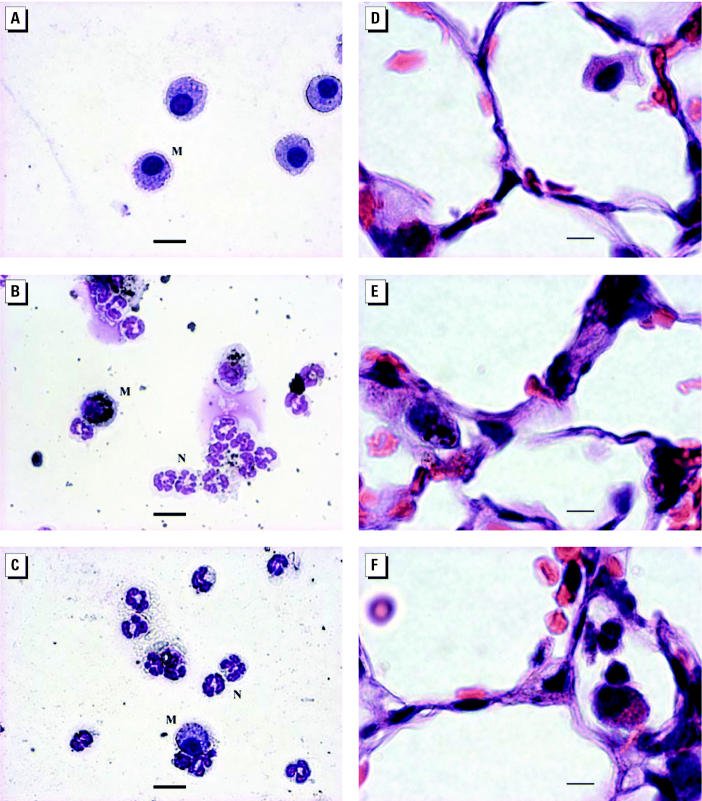
BAL cytology (Liu stain; *A*–*C*) and histopathology (H&E stain; *D*–*F*) at 16 hr after instillation of ufCB (200 μg/mouse) or LPS (100 μg/mouse). (*A*) PBS control; mainly macrophages. (*B*) ufCB group; mainly neutrophils. (*C*) LPS group; mainly neutrophils. (*D*) PBS control; intact alveolar wall. (*E*) ufCB group; thickened alveolar wall, macrophages with ufCB aggregates. (*F*) LPS group; positive control. Abbreviations: M, macrophage; N, neutrophil. Bars: *A*–*C*, 15 μm; *D*–*F*, 5 μm.

Figure 3 presents illustrations of cytospins and histopathologic pictures from mice after 16 hr of exposure to PBS, ufCB (200 μg/mouse), or LPS (100 μg/mouse). Macrophages appeared as giant cells with nonsegmented nuclei. Neutrophils had segmented nuclei and were smaller than macrophages. In ufCB and LPS treatment groups, most inflammatory cells were neutrophils (Figure 3B,C). ufCB aggregates could be seen in macrophages (Figure 3B,E).

Histopathologic study revealed thickened alveolar walls in the lungs of mice that received ufCB treatment (Figure 3E). There were less obvious changes in mice challenged with LPS (Figure 3F). The alveolar wall remained intact in PBS treatment group (Figure 3D).

### Cytokine production and alveolar-capillary permeability.

**Figure 4:**
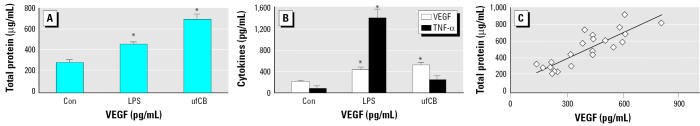
Correlation of VEGF with total proteins after 16 hr of exposure to ufCB (200 μg/mouse) or LPS (100 μg/mouse). (*A*) Total proteins. (*B*) VEGF and TNF-α . Data are expressed as means ± SE of eight mice (two separate studies, each with *n* = 4). (*C*) Regression analysis; significant correlation between VEGF and total proteins (*R*^2^ = 0.7352, *p* < 0.01).
*Significantly different from the control group (*p* < 0.01).

Compared with PBS control, ufCB caused a significant increase in protein contents at 16 hr postinstillation, as did the LPS treatment group (*p* < 0.01) (Figure 4A). ufCB and LPS both triggered significant increases in VEGF productions, compared with PBS control (*p* < 0.01) (Figure 4B). In contrast to the significant increase of TNF-α in LPS treatment group (*p* < 0.01), the TNF-α level of ufCB treatment group was not substantially different from that of the PBS control group at 16 hr postinstillation (Figure 4B).

To explore the role of VEGF in ufCB-induced protein leakage, we plotted the total proteins against the VEGF, using the data from Figure 4A,B. Analysis showed a coefficient of determination (*R* ^2^) of 0.7352 between VEGF and total proteins (*p* < 0.01; Figure 4C). The data strongly suggested that the secretion of VEGF120 and −164 was associated with the increase of alveolar-capillary permeability.

### The involvement of ROS in ufCB-induced production of VEGF in mice.

**Figure 5:**
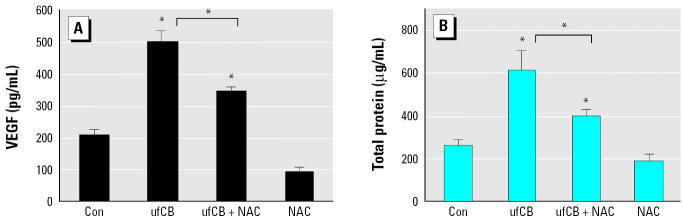
Effect of NAC (320 mg/kg, 2 hr before instillation) on (*A*) VEGF productions and (*B*) total proteins in mice after 16 hr of exposure to ufCB (200 μg/mouse). Data are expressed as means ± SE of four mice.
*Significantly different from the control or between the ufCB + NAC group and the ufCB group (*p* < 0.01).

The effects of interventions with NAC on VEGF productions and total proteins in mice are shown in Figure 5A,B. Pretreatment with NAC (320 mg/kg) 2 hr before ufCB instillation significantly lowered the levels of VEGF and total proteins, compared with ufCB treatment alone (*p* < 0.01). These findings supported the role of ROSs in ufCB-induced VEGF release and protein leakage.

### The involvement of ROS in ufCB-induced production of VEGF in cultured cells.

**Figure 6:**
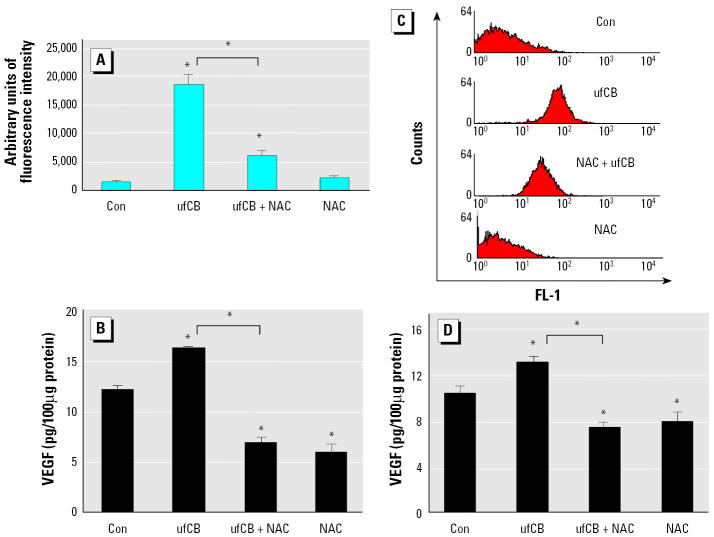
Effect of NAC (20 mM, 1-hr pretreatment) on oxidative stress and cellular VEGF production in cells treated with ufCB (100 μg/mL) for 4 hr. (*A*) Oxidative stress in THP-1 cells. Data are expressed as means ± SE of four or five separate experiments; ufCB group, *n* = 5. (*B*) Cellular VEGF in THP-1 cells. (*C*) A representative histogram in which the fluorescence (FL-1) is plotted against the number of A549 cells. Ratios of mean fluorescence intensity of treated cells to PBS control: control (Con), 1; ufCB, 11.6; NAC + ufCB, 6.19; NAC, 0.71. (*D*) Cellular VEGF in A549 cells. Data are expressed as means ± SE of four (THP-1) or three (A549) separate experiments.
*Significantly different from the control or between the ufCB + NAC group and the ufCB group (*p* < 0.01).

To strengthen the hypothesis that oxidative stress played a role in ufCB-induced production of VEGF, the effects of NAC on ROS and VEGF productions were analyzed in ufCB-treated THP-1 and A549 cells. ufCB at 100 μg/mL triggered a significant increase of ROSs in THP-1 cells, as measured by the increase of DCF fluorescence intensity. Pretreatment with NAC (20 mM) 1 hr before ufCB exposure significantly reduced ROS production, compared with ufCB treatment alone (*p* < 0.01) (Figure 6A). The addition of NAC also significantly prevented ufCB-induced VEGF production in THP-1 cells, compared with ufCB treatment alone (*p* < 0.01) (Figure 6B). Figure 6C shows a representative histogram in which the fluorescence is plotted against the number of cells. ufCB at 100 μg/mL caused the oxidative stress in A549 cells, as indicated by the ratio of mean fluorescence intensity of ufCB-treated cells to PBS-treated control cells (ratio = 11.6). Pretreatment with NAC 1 hr before ufCB exposure lowered the level of ufCB-induced oxidative stress, as indicated by the ratio of mean fluorescence intensity of ufCB plus NAC-treated cells to PBS-treated cells (ratio = 6.19). The addition of NAC significantly reduced ufCB-induced VEGF production in A549 cells, compared with ufCB-treated cells without antioxidant intervention (*p* < 0.01) (Figure 6D). Taken together, exposure to ufCB caused the oxidative stress, and NAC prevented ufCB-induced oxidative stress and production of VEGF in THP-1 and A549 cells. These data indicate that oxidative stress plays a significant role in ufCB-induced VEGF production.

## Discussion

Previous studies of ufCB have focused on its proinflammatory effects, including the influx of polymorphonuclear cells and the induction of proinflammatory cytokine TNF-α from cultured alveolar macrophages (Dick et al. 2003; Donaldson et al. 1998; Li et al. 1999). Our data in Figure 2A reveal a peak increase of TNF-α in BAL fluid 4 hr after instillation of ufCB. TNF-α has been shown to be elevated in the early phase of LPS-induced acute lung injury. An 8-hr, but not 16-hr, exposure to heat-killed bacteria induces the maximal increase of TNF-α in BAL fluid and the peak increase in epithelial permeability, as measured by the appearance of instilled I^125^-BSA in the blood. Moreover, inhibition of TNF-α by antibodies against TNF-α can prevent heat-killed bacteria-induced epithelial permeability (Li et al. 1995). In a murine model of acute lung injury, significant increases in TNF-α appear in BAL fluid within 2 hr after instillation with LPS, but TNF-α levels return to the basal level by 24 hr postinstallation (Karmpaliotis et al. 2002). The permeability effect of TNF-α has been attributed to alterations of the tight junction meshwork in studies using human epithelial monolayer cells (Gitter et al. 2000; Schmitz et al. 1999). TNF-α can also reduce integrin-mediated adhesion to fibronectin and disrupt lung endothelial integrity (Rotundo et al. 2002). Therefore, the increased protein leakage 4 hr after instillation of ufCB in the present study may be caused partly by the production of TNF-α

Because of the large number of particles and the large surface area per unit mass, a sub-stantial portion of ultrafine TiO_2_ and ufCB can easily evade macrophage clearance. This prolongs particle retention time, increases interactions between particles and epithelial cells, and leads to the oxidative stress of epithelial cells (Donaldson et al. 1998; Ferin et al. 1992). The increased oxidative stress reduces epithelial cell integrity. Breaches in the epithelial barrier facilitate the transfer of ufCB to the interstitial area and airway smooth muscle. *In situ* hybridization studies show that VEGF mRNA is mainly expressed in the alveolar epithelial cells (Bhatt et al. 2000; Maniscalco et al. 1995). In addition, airway smooth muscle and macrophages are other potential sources of VEGF (Cho et al. 2001; Fehrenbach et al. 1999). The prolonged interactions with a variety of target cells provide a reasonable explanation as to why a single challenge of ufCB causes sustained increases of VEGF in BAL fluid even at 42 hr postinstillation (Figure 2C).

VEGF activates VEGF receptor-2 (VEGFR-2/Flk-1/KDR) and increases capillary permeability by tyrosine phosphorylation of the interendothelial adhesion molecule VE-cadherin (Esser et al. 1998). Through its interaction with VEGFR-2 in the endothelial cells, VEGF also enhances the production of nitric oxide and prostacyclin and increases vascular permeability (Murohara et al. 1998). A single VEGF gene gives rise to three protein isoforms (VEGF120, VEGF164, and VEGF188) through alternative mRNA splicing in mice (Shima et al. 1996). In mouse lung tissues, RNase protection assay indicates that the most abundant isoform mRNA is VEGF188 mRNA and the least abundant isoform is VEGF120 mRNA (Ng et al. 2001). However, little attention has focused on the protein abundance of different VEGF isoforms in the induction of alveolar-capillary permeability. Our data indicate that ufCB causes a sustained increase of secreted VEGF in BAL fluid (Figure 2C). The expression of cell-bound, nonsecreted VEGF188 is not altered in Western blotting analysis (Figure 2B). As a result, the increased VEGF120 and VEGF164 isoforms are associated with the alveolar-capillary permeability.

Normal human lungs express higher levels of VEGF189 relative to VEGF165 and VEGF121. In non-small-cell lung carcinoma, mRNA analysis has shown predominant expressions of VEGF121 and VEGF165, when compared with normal lungs (Cheung et al. 1998; Fontanini et al. 1999). The soluble isoforms are related to poor prognosis and metastasis (Fontanini et al. 1999; O’Byrne et al. 2000). Different VEGF isoforms have distinct functions in tumor angiogenesis. In mice, the most diffusible VEGF120 can recruit vessels at some distance from the secreting cells. The nonsecreted VEGF188 increases the concentration locally and works cooperatively with VEGF120 to expand the capillary bed. VEGF164 is capable of recruiting vessels with its partial diffusible property and vascularizing the tumor with its partial nonsecreted property. Therefore, VEGF164 alone can enhance tumorigenic vascularization and tumor growth (Grunstein et al. 2000). Further understanding of the regulatory mechanism controlling differential expression of VEGF isoforms after exposure to ufCB may have great implications for our knowledge of the pathogenesis of lung carcinoma.

As indicated above, results from instillation studies with ufCB have been controversial regarding the coincidence of neutrophils influx and the increase of protein leakage in rats (Dick et al. 2003; Li et al. 1999). In our study with mice, the influx of neutrophils is not significantly evident until 16 hr after instillation (Figure 1B). The increase in protein leakage occurs without the presence of significant influx of neutrophils in the early phase of ufCB-induced acute lung injury. It is intriguing to note that ufCB induces a maximal increase of VEGF at 16 hr, which occurs before the peak increases of neutrophils influx and protein leakage at 21 hr in our study (Figures 1A,B, 2C). Leukocyte adhesion to endothelial cells is the step proceeding leukocyte infiltration, because leukocyte adhesion can trigger disorganizations of endothelial cell adherens and tight junctions and increases in vascular permeability (Del Maschio et al. 1996; Kurose et al. 1994). VEGF can induce the expression of adhesion molecules that bind leukocytes to endothelial cells (Detmar et al. 1998; Kim et al. 2001). Furthermore, VEGF has been demonstrated in activated neutrophils (Gaudry et al. 1997). Neutrophils, via their own VEGF, can further enhance vascular permeability when they bind to endothelial cells. Therefore, the increase in neutrophil adhesion and infiltration may be another mechanism in the generation of VEGF-induced vascular permeability.

Studies with deletion mutants of VEGF promoters have shown that H_2_O_2_ increases macrophage VEGF production through activation of the VEGF promoter, located between –449 and –1 (Cho et al. 2001). A recent report has demonstrated that the oxidative stress incurred by H_2_O_2_ increases the *VEGF-A* gene expression by increasing the binding of transcriptional factors Sp1 and Sp3 to proximal GC-rich motif in AGS human gastric adenocarcinoma cells (Schafer et al. 2003). In our study, the addition of NAC blocks the ufCB-induced increase of VEGF in mice (Figure 5A) and prevents the generation of ROS and VEGF in THP-1 and A549 cells (Figure 6). These support the involvement of ROS in ufCB-induced production of VEGF in lung tissue. Further study is warranted to investigate the signal transduction pathway responsible for ufCB-induced VEGF gene expression in epithelial cells.

In conclusion, the peak increase of VEGF appears before the maximal increases of neutrophils influx and total proteins. Protein leakage is highly correlated with VEGF production. Interventions with NAC can reduce VEGF productions and protein leakages in ufCB-treated mice. The involvement of ROS is strengthened by the fact that NAC prevented ufCB-induced generations of ROS and VEGF in THP-1 and A549 cells. This is the first report to demonstrate the ROS-dependent induction of VEGF by ufCB and the involvement of VEGF in ufCB-induced lung injury. With the capability of escaping macrophage clearance, ufCB has great opportunity to cause profound effects. Future studies will focus on the health effects of long-term exposure to ufCB.
